# Medical Items and News of Interest to the Physician

**Published:** 1878-07

**Authors:** 


### For the Use of Physicians.

CULLED FROM THE STANDARD MEDICAL JOURNALS OF THE WORLD.

NOTE.—These formulæ are intended only for the regular practitioner of medicine, and should be employed only by him, or under his immediate supervision.

**Deodorized Iodoform.**

Dissolve in ether and apply to the diseased parts. On evaporation an odorless coating of iodoform is left.

**SclerotinicAcid for Hypodermic Injection.**

Professor Dragendorff says that sclerotinic acid, which is separable by means of alcohol, is a more powerful and less irritating remedy for hypodermic injection than ergotin. [The latter is at best but an uncertain product].

**Pepsine from the Ostrich's Stomach.**

According to the *Revue des Deux Mondes*, the ostrich hunters of South America, bearing in mind the almost incredible digestive powers of that bird, extract the pepsine from its stomach, and sell it for its weight in gold to dyspeptics.

**Laboratory Vintage.**

The chemists of Berlin have been occupied lately in analyzing the wares of the wine merchants, and no little excitement has been caused by the discovery that the entire stock of one of the largest houses dealing in wines for medicinal purposes, consisted entirely of artificially prepared mixtures of spirit and sugar solutions, flavored with various herbs.

**Arsenical Poisoning.**

James Hayes, M. D., of Simcoe, Ont., publishes, in the *Canada Lancet* for March, a case of arsenical poisoning treated with Wyeth's dialyzed iron. Following an emetic and free draughts of warm water, a teaspoonful of the dialyzed iron was given and soon ejected; doses of thirty drops were then given every twenty minutes for two hours. Two hours after the doctor's arrival, symptoms of collapse set in, and were treated with brandy, hot bottles, and friction. The patient was restored to health in about ten days, and complained during convalescence of weakness, thirst, and a burning sensation in the stomach. The doctor estimates that fully a teaspoonful of arsenious acid was lying in the stomach from half an hour to an hour before he saw her.

**Chorea.**

Prof. H. C. Wood suggests a saturated tincture of the rhizome of *Dracontium fœtidum* given in doses of 60 to 90 drops, three times daily, as a remedy for chorea. It must be made from fresh roots collected in the fall and before drying.

**For Prurigo.**

℞

Acidi carbolici, ʒ j.

Acidi hydrocyanici, ʒ j.

Morph. sulph, gr. vj.

Hyd. chlor. corros, gr. vj.

Glycerini, ℥ iij.

Misce. S. Apply to the itching part.

**For Painful Hæmorrhoids.**

℞

Liquor bismuth, ℥ j.
Tinct. opii. deodorat, ʒ j.
Mucilag. amyli, ℥ iij.

M.

Sig.—Inject one-fourth every three or four hours.—*Louisville Medical News.*

**For Dysentery.**

℞

Bismuth subnit, ℥ ss.
Acid. salicylici, gr. vj.
Acid. carbolici, ♏ iij.
Tinct. opii (Sydenham) fl. ℥ j.
Tinct. belladonnæ, fl ℥ j.
Aquæ, O j.

M. S. Inject one ounce with baby-syringe after each evacuation.—*Canadian Jour. of Med. Science.*

**Acute Articular Rheumatism.**

M. I. Guerin read a paper at a meeting of the Paris Academy of Medicine, on the 4th of September, on the abortive treatment of acute articular rheumatism by means of what he terms the stibiodermic method. This consists in the inunction every six or eight hours of an ointment containing one part of tartar emetic in two of lard. He states that a rapid reduction may thus be obtained of the pain experienced in the early stage of coxalgia. The cessation of the pain he attributes to a dynamic action of the remedy, and not to any revulsive action. The same results were obtained in gout when the symptoms only preluded the definitive attack, three or four inunctions being sufficient to prevent the onset of the disease. When, however, the attack was fully developed, a flying blister, placed on the centre of the swelling, quickly effected its removal.—*Archives generale de Medicine.*

**Large Dose of Chloral.**

James J. Healey, M. D., of Newburyport, Mass., reports the case of a man, aged forty, suffering from over drinking during a period of two weeks, and who, through disobedience of a nurse, took, in the course of ten hours, ten drachms of chloral-hydrate, with the effect only of producing five or six hours of restless sleep.—*Boston Med. and Surg. Jour.*

---

**Nitric Acid for Whooping Cough.**

Take of Nitric acid, dil. fl. ʒ. xii

Comp. tincture of cardamoms fl. ʒ. iij.

Syrup fl. ℥. iij. ss.

Water fl ℥. j.

Mix. One to two teaspoonfuls every two hours, according to the age of the child. This is said by Sir G. D. Gibb to be a specific, and will cure in from two days to two weeks.

---

**Ointment for Hæmorrhoids.**

℞

Acidi tannici, ℈j.

Acidi carbolici, gr. v.

Ext. opii., ℈j.

Ext. bellad, } āā ℈ ss.

Ext aconiti, }

Cerat. simp., ℥ j.

Misce bene. S. Apply to the piles morning and evening, and introduce a small piece into the bowel after each action.

---

**Electuarium Sennæ Compositum.**

| | | |
|---|---|---|
| Barley | 60 | parts. |
| Polypody root | 60 | " |
| Licorice | 30 | " |
| Hart's tongue root (Scolopendrium) | 45 | " |
| Mercury plant (Murcurialis annua) | 120 | " |
| Dry raisins | 60 | " |
| Powd. senna leaves | 150 | " |
| " fennel | 10 | " |
| " anise | 10 | " |
| Jujube | 45 | " |
| Senna leaves, whole | 60 | " |
| Cassia pulp | 200 | " |
| Tamarind pulp | 200 | " |
| Prune pulp | 200 | " |
| Sugar | 1200 | " |

Boil the barley in water, then add the polypody and finally the licorice, hart's tongue, mercury plant and the raisins. Strain and express. Make a dilute decoction of the senna, and add the liquid to the former; then evaporate the whole to 2,500 parts. To this add the sugar, so as to form a syrup, and finally incorporate with it the fruit pulps and the powders.

**Fuller's Tamarind Electuary.**

| | | |
|---|---|---|
| White sugar | 10 | parts. |
| Manna | 25 | " |
| Boiling water | 50 | " |

Dissolve and filter. When cold add

| | | |
|---|---|---|
| Tamarind pulp, purified | 15 | parts. |
| Potas. bitartrate | 1 | " |
| Senna leaves, powd. | 4 | " |

The finished product should weigh 100 parts.

---

**Incontinence of Urine.**

Extr. nucis vom. alcoh., (4 grains.)

Ferri phosphat. (protoph.) (46 grains.)

Extr. quassiæ, (31 grains.)

Rad. Gentianæ, q. s.

M. 25 pills.

One pill three times a day, in conjunction with cold hip bath, and abstention from drink during the evening.

---

**Sedative Pills, Gunther's.**

These are composed of the following ingredients:

Asafœtida, powd., 50 parts.
Extract of valerian, 50 parts.
" belladonna, 3 parts.
Oxide of zinc, 1 parts.
Castor, 2 parts.

Make into a pill-mass, to be administered in doses of 3 to 10 grains, twice daily, in chorea, etc.

---

**Lotion for Sore Nipples.**

A correspondent of the *American Practitioner* gives the following as the lotion for sore nipples used in the famous "Rotunda" Lying-in Hospital of Dublin.

℞

Powdered borax, ʒ ij.

Powdered chalk, ʒ i.

Spirit of wine, ℥ ij.

Water, ʒ ij.

M.

---

**Abscess of the Liver.**

What will this acid not do? It is not good, probably, for all sorts of human ailments, but it is useful in many. Dr. Sheehy, in the *British Medical Journal*, relates a very interesting case, which had only grown worse under other approved treatment, that was cured rapidly by inhaling carbolic acid, half a drachm in half a pint of hot water, for ten minutes each time, twice a day. The enormous purulent expectoration began to abate after a few days, and at the end of two weeks the patient had passed from a most critical condition, and was able to go abroad and face all kinds of weather.

**Neutralizing Poison with Sweet Oil.**

A poison of any conceivable description and degree of potency, which has been intentionally or accidentally swallowed, may be rendered almost instantly harmless by simply swallowing two gills of sweet oil. An individual with a very strong constitution should take nearly twice the quantity. This oil will most positively neutralize every form of vegetable, animal, or mineral poison with which physicians and chemists are acquainted. —*Journal of Applied Science.*

---

**Bossu's Laxative Mixture.**

Resin of scammony.
" jalap, āā ½ grain.
Croton oil, 2 drops.
White sugar, 15 grains.
Mucilage, 25 grains.
Orange flower water, 4 ℈
Compound syrup of senna, 1 fl. ℥
Peppermint water, 3 fl. ℥

The first five ingredients having been properly triturated together and emulsionized, the remainder is added carefully. Dose one tablespoonful.

---

**Arsenate of Gold.**

Extraordinary powers have lately been attributed to arsenate of gold, as a remedy in nervous affections of every variety. It may be prepared by mixing 1.8 grammes of chloride of gold and sodium (Germ. Pharm.) with 0.55 grammes of neutral, dry sodium arsenate, rubbing the mixture with 1 gramme of water, and drying again at a moderate temperature. From the published reports it would appear that the remedy had also been used in trituration, by homœopathists, and as the dose would naturally have to be quite small, such a method of administration would probably be preferable.

---

**Dialyzed Iron Hypodermically.**

Professor Da Costa has employed dialyzed iron in a novel manner, in a case of chlorosis, in a woman aged twenty-one years. The patient had daily injections of fifteen minims of the iron solution, at first diluted, but afterwards of full strength. The points where the injections were made showed no evidence of inflammatory action. Subsequently the dose was raised to 30 minims, and convalescence was rapid. After the hypodermic use was ceased, 20 drops in water were given thrice daily for a short time. No constipation, indigestion, or other disturbances resulted from this mode of using the remedy, and recovery was considered to have been more rapid than it would have been with the usual way of administering it.

**Hoarseness.**

Dr. Eyselin (see *The Practitioner* of February) gave an infusion of 45 grains of jaborandi leaves to a patient suffering with hoarseness and pain in the neck, due to a cold. The effects of the remedy were soon manifested, profuse perspiration continued for two hours and soaked the bed-linen. Salivation was also marked. The hoarseness disappeared during the sudorific action, but chills occurred two or three times during the same period, and the patient in the end complained of weakness and lassitude. Next day the left submaxillary gland was enlarged and tender, the appetite poor, and the saliva thick. The effect on the hoarseness was all that could be desired, but it was attained at a cost of very great discomfort.

---

**Diabetes Insipidus.**

Prof. Da Costa's clinic on January 12th exhibited a patient who had been passing ten pints of urine daily, the amount of fluid ingested being fully two pints less. No cause could be assigned for the disease. The urine contained no sugar or albumen, and had a s. g. of 1,005. When commencing the use of ergot, drachm-doses of the fluid extract were taken three times daily, then four times, and at last six times daily. The effect on the renal secretion was to reduce its amount to five pints, then to three pints, by which time the ergot had been stopped and peppermint water substituted. During two weeks of this supplemental treatment there was no return of the disease. Prof. Da Costa has had three other cures by this method, one which was verified a year after its discharge.—*Med. and Surg. Rep.*

---

**Aromatic Syrup of Licorice.**

℞

Powd. ext. of licorice, ℥ jv.

Jamaica ginger, cinnamon bark, each, ʒij.

Cloves, ℥ j.

Sugar, ʒlx.

Water, *ad*, Ojv.

Reduce the cinnamon, ginger, and cloves to a coarse powder, and boil in two pints of water over a slow fire for one hour. Then strain, and dissolve in the liquid the powd. ext. of licorice, with the aid of a gentle heat, stirring to assist the solution. When dissolved add the sugar, keeping up the heat till the latter is also dissolved; then strain while hot, and add hot water through the filter to make four pints of finished syrup.

This is recommended as a most effectual disguise to the taste of quinine.—*Am. Jour. Pharmacy.*

**Eruption by Poison Oak.**

Dr. S. A. Brown, U. S. N., writes to the *Medical Record* that he has found in bromine a specific to the eruption produced by the poison oak or poison ivy. He says that he has used it with unvarying success in at least forty cases. The eruption never extends after the first thorough application, and it promptly disappears within twenty-four hours if the application is persisted in, and the patient is entirely cured. He uses the bromine dissolved in olive oil, cosmolin, or glycerin, in the strength of from ten to twenty drops of bromine to the ounce of oil, and rubs the mixture gently on the affected part three or four times a day. The bromine is so volatile that the solution should be renewed within twenty-four hours from its preparation.

---

**R. Rother's Formula for Bay-Rum.**

True bay-rum is made from *Pimenta acris*, and not from *Laurus nobilis*, as commonly supposed; the method of its distillation not being known outside the West Indies, it has been customary to make it extemporaneously with the oil of bay distilled from the leaves of the former plant. This preparation is inferior in fragrance, however, to the genuine article. The following formula of R. Rother is said to give very good results: Take of

Oil of bayberry, 1 fluid ounce.
Jamaica rum, 1 pint.
Strong alcohol, 4 pints.
Water 3 pints.

Mix the rum, alcohol, and water, then add the oil; mix and filter.

---

**Chronic Pleurisy.**

Professor Da Costa in his clinic (see *Med. and Surg. Rep.* of Feb. 23d) showed a man, aged 23 years, who had chronic pleurisy since last summer, and had been under treatment at the Episcopal Hospital, when his chest had been aspirated, and five pints of clear serum removed. Five weeks afterwards, he had a return of chills, which had troubled him daily previous to the operation, and an examination showed a re-collection of fluid. As an alternative to aspiration, Dr. Da Costa, on the patient's admission to the Pennsylvania Hospital, gave him a drachm of jaborandi, four times daily. At the end of a week, under the influence of the remedy, the chills passed away, likewise the accumulation of serum in the pleural cavity, the temperature fell from 101° to 98° and 99°, and the patient ultimately recovered. Profuse sweating continued during the action of the remedy.

**Ergotine Suppositories.**

Mr. Connolly, at an evening meeting of the Pharmaceutical Society of Ireland, communicated the following formula for making ergotine suppositories. They are very generally used by practitioners in Ireland:

Hard soap, 1 drachm.
Water, 30 minims.
Ergotine, 32 grains.
Glycerine, ½ drachm.

Melt the soap and the water, with a gentle heat, and add the glycerine; evaporate to get rid of the water, add the ergotine, and pour into moulds.

By this manipulation a nice suppository is obtained which is difficult to make with glycerine alone.

---

**Soluble Medicated Intra-Uterine Bougies.**

Since M. Reynal, of Paris, introduced this method of applying remedies to the canal of the urethra and uterus, it has grown quite popular, and the manufacture of the bougies has been undertaken by a considerable number of persons. Mr. Charles L. Mitchell, of Ninth and Race Streets, Philadelphia, has recently introduced a line of these goods of which he furnishes samples containing two grains of zinc acetate, and a list of twenty other varieties, all specially adapted for the treatment of intra-uterine affections. We are very well pleased with the appearance of the samples received, and from the experience we have had in using similar preparations, can confidently recommend their use.

---

**Chloral and Opium.**

T. S. Shields, M. D., of Crawfordville, Ga., reports (*Southern Medical Record*) the case of a woman who had been taking daily 40 to 60 grains of chloral before she came under his care, and who subsequently became habituated to the use of opium. She was subject to attacks of hysteria, against which the commonly used remedies were employed. In an attack characterized by active delirium, wakefulness with a pulse of 120, respiration 24, and no fever, 10 grains of chloral in water were administered. An hour or two before, the patient had taken 6 grains of gum opium. A quarter of an hour after the chloral was taken, symptoms of opium poisoning supervened, and in a very short period progressed to complete arrest of respiratory movements. After great efforts, respiration was restored by means of caffein, atropia, and galvanism. Since the patient had been accustomed to the use of opium, the writer attributes to the chloral the intense effect produced in this instance.

**Local Antiseptic.**

Professor J. A. Larabee, of Louisville, having lately had an experience with puerperal feyer in the Forest Hill Lying-in Hospital, believes that carbolic acid, although much lauded, is over-estimated, is entirely unreliable as a disinfectant, and merely substitutes its own odor for that of the disease. On the other hand he believes that a solution of mild strength of chloral in water, used with a douche or fountain syringe, not only removes the odors of the lochial discharges, but destroys the noxious influences of the poisons it contains. At the outset, four deaths took place within a brief period under the carbolic acid treatment. After the resort to chloral injections in every case, thirty-nine deliveries took place previous to the date of the communication, without an untoward result.—*Louisville Med. News.*

---

**Delirium Tremens.**

J. Farrar, of Edinburgh, failing in a severe case of delirium tremens after two or three days of treatment with opiates, resorted to chloral hydrate as follows: At 5:10 A. M., thirty grains, a like quantity half an hour later. At 6 A. M., a hypodermic dose of ½ grain of morphia. At 6:10 forty grains of chloral were given. At 6:25 ⅜ grain of morphia was injected, and at 6:55 the patient was asleep, and so remained for eight hours. Recovery followed at once.

In another case, in which digitalis, morphia, etc., had been ineffectual, chloral was resorted to in doses repeated every ten minutes until 160 grains had been taken. Seven hours sleep then took place, and was followed by recovery.

During the first two doses the patient was more excited and garrulous—indeed *intoxicated*, but this soon gave way to thick speech, inarticulate mumblings, and then sleep.—*Br. Med. Jour.*

---

**Sulphuric Acid Beer.**

Take of Molasses 14 pounds.

Bruised ginger, ½ pound.

Coriander, ½ ounce.

Capsicum, ¼ ounce.

Cloves, ¼ ounce.

Water (hot) 12½ gallons.

Yeast, 1 pint.

Macerate with frequent stirring in a covered vessel, and when cool, add the yeast; keep in a moderately warm situation to excite active fermentation. The next day, decant the liquor and strain it through flannel, and allow the fermentation to continue for a couple of days longer; then add of *sulphuric acid*, 1½ ounce (diluted with eight times its weight of water), and of *bicarbonate of soda*, 1½ ounce (dissolved in a little water). In two or three days more it will be fit to use. This furnishes a pleasant occasional drink for persons engaged in making white lead, for painters, and others employed in occupations in which lead is used. It is said to be a preventive of lead colic, and is advantageous as a curative agent. Another drink known as "sulphuric acid lemonade" is also advised for a similar purpose.

---

**Cod Liver Oil with Hypophosphites and Whiskey.**

The following makes a nice and palatable emulsion:

Ol. morrhuæ, 4 fl. ℥.

Pulv. acaciæ, 2 ℥.

Syrup pruni virg, 2 fl. ℥

Spts. frumenti, 1½ fl. ℥.

Calcii hypophosph.,

Sodii hypophosph, āā 1 ʒ.

Ol. gaultheriæ, 24 ♏.

" amygdalæ amar, 10 ♏.

Aquæ. q. s. ad, 12 fl. ℥

Dissolve the hypophosphites in the water. Rub the powdered gum arabic with a little of the water to a paste, then add a small quantity of the cod liver oil, and triturate thoroughly. Again add a little of the water, and then some of the oil, alternately, under constant trituration, until they are thoroughly emulsionized. Dissolve the essential oils in the whiskey, mix this with the syrup, and incorporate the latter with the emulsion first formed.

---

**The Use of Ergot in Cases of Sumucous Uterine Fibroid.**

The use of ergot as a means for producing the atrophy of myo-fibromata of the uterine walls, has become quite the accepted mode of treatment. In the *Medical Times*, of Philadelphia, Henry Brubacker, of Somerset, Pa., reports two cases in which this mode of treatment was successful. The first had long suffered from menorrhagia, and on careful examination was found to have a fibroid the size of a fœtal head, attached to the fundus and left side of the uterine walls, and submucous in character. Repeated five grain doses of ergotine hypodermically caused decided uterine contractions, which resulted in sloughing and extrusion of the tumor, the growth being discharged piece-meal. The last portion was ligated by means of a Gooch's double canula. The second case resembled the first, so far as relates to the nature of the growth, but the fluid extract of ergot was given by the mouth in one-ounce doses daily. About four weeks of treatment sufficed to cause the mass to be squeezed into the vagina, where it was readily ligated and removed. No symptoms of septicæmia occurred in either case.

**Epistaxis.**

Dr. Blondeau relates, in the *Union Medicale*, a case of epistaxis in which a large quantity of blood had been lost, and which, not yielding to other means, he succeeded in rapidly arresting by the application of a tape tightly around the middle part of the thigh. As the bleeding did not recur this was removed next day, when the hæmmorrhage reappeared soon after. A new application of the ligature was made, and was again promptly successful, the bleeding again returning when it was removed. At last, after a series of these alternations, the hæmmorrhage ceased entirely.—*Med. and Surg. Rep.*

**Elimination of Mercury.**

Dr. E. W. Hamburger, of Franzensbad, sums up a paper in the *Prager Medicin. Wochenschrift,* with the following conclusions: 1. Mercury may be distinctly found in the urine of patients who have been treated with mercurial suppositories for some time. In one case in which the treatment had been commenced four days previously, mercury was not found in the urine. Mercury is always present in the urine of patients who have been treated by inunction.

2. In patients treated by suppositories, mercury was always found in the milk as well as the urine. In cases of inunction, although mercury was present in the urine, none could be found in the milk; and, when mercurial inunction was substituted for suppositories, the mercury disappeared from the milk, although it continued to be present in the urine.

3. The fæces of a patient, who was treated by innuction, contained a large amount of mercury. Dr. Hamburger concludes hence that the elimination of mercury takes place chiefly by the bile.

The chemical process used consisted in the removal of organic matters, the application of electrolysis, and the formation of iodide of mercury, the crystals of which were readily recognizable under the microscope.—*London Med. Record.*

**Typhoid Fever.**

Dr. Perrse, Surgeon to the Meath Hospital (Dublin), has had long experience with turpentine in typhoid fever. He claims to have had with it great success. This oil has had in all ages some reputation. It acts as a stimulant, diuretic, diaphoretic, and laxative if the dose is sufficient, and it is a first-class anthelmintic. Its use in typhoid fever is by no means new; but it has been perhaps too much neglected by the medical profession. It is too common! The following is Dr. Perrse's prescription; it has a judicious appearance. He says:

"My mode of giving the turpentine was as follows. If bronchitis were present, and even if diarrhœa complicated the case, I gave what was known as my turpentine mixture—

℞

Terbinthinæ olei. ʒ ij.

Liquoris potassæ, ʒ ij.

Mucilaginis acaciæ, ʒ iv.

Syrupi papaveris albi,

Syrupi floris aurantii, āā ʒ viij.

Aquæ camphoræ, q. s. ad. f ℥ viij.

Fiat mistura. A tablespoonful to be taken every fourth hour, the bottle being first shaken.

Since I commenced that treatment, I have never lost any case of typhoid, from either bronchitis or diarrhœa, or from its sequelæ of ulceration or hemorrhage.

**Ingrowing Toe-Nail.**

Drop a few drops of liq. potassæ ( ʒ iij. to ℥ j., as recommended by Dr. Sidney Ringer) on the ulcerated surface, with its imbedded nail, four or five times a day, and you will soon notice a diminution of the discharge, a cutting-down of the granulations, and the edge of the nail beginning to pulpify. Here I am met with the objection that this will give rise to intolerable suffering. The pain, acute at first, will soon pass away, and leave the patient much more comfortable than before. Practicing this for a short time, a partly-flexible condition will be observed in the inverted nail, which renders it with proper care and caution, easily elevated, without causing the patient too much pain. When this is done, take a thin piece of selected cork, which, being smooth, flexible, and capable of accommodating itself to this bed of granulations, is gently inserted under the nail, to be followed by almost instantaneous relief. Compressed sponge, lint, and cotton wool are all open to a common object, viz., their properties of absorbing the discharge from the granulations and retaining it in contact with the sore, which is highly detrimental to its favorable course and cure. They are, moreover, too heating, and not sufficiently firm and elastic to exercise the necessary degree and kind of pressure. These latter indications are fulfilled most admirably and perfectly by the cork, causing by constant pressure atrophy and wasting of the granulations and the great mass of redundant tissue. Aside from these properties of the cork, by constantly elevating the nail it separates to an appreciable extent the nail from its matrix, thus causing in the course of a few months a marked narrowing of the nail.—J. D. NEET, in *Medical Record.*

---

**Rheumatism.**

E. E. Milam, M. D., of Paris, Tenn., reports, in the *Louisville Med News* of Feb. 16th, six cases of rheumatism in which the salicylate of soda was employed. The first, a little girl of eight years, had a swelling of the wrists, elbows, and knees. Salicylic acid, in doses of ten grains, every four hours, produced no effect, and, except when under the influence of morphia, the pain was intense. Death occurred on the tenth day. In the second case, a girl of seven years, had rheumatic inflammation of the left elbow and wrist. Salicylate of sodium, in doses of fifteen grains every four hours, produced an amelioration of symptoms within six hours, and recovery took place within two days. In case third, the curative effect of the salicylate of sodium was equally marked. Case fourth had been sick with acute rheumatism for two weeks, when 30 grains of salicylate of sodium were ordered to be taken every four hours; the pain was as effectually relieved as if opium had been used, and at the next visit the doctor found her almost relieved of the symptoms of rheumatism. At the end of the third day from the commencement of the treatment, only tonics were necessary; a relapse a short time afterwards was as quickly subdued. The fifth case, a colored woman, had severe rheumatism, and was relieved of most of her symptoms within two days, by doses similar to those employed in case fourth. One wrist remained swollen and was treated with stimulating liniments in addition to the sodium salicylate. By the sixth day she, too, had entirely recovered. The sixth case, though somewhat more tedious, showed likewise the favorable results of the use of the remedy. In summing up, Dr. Milam indorses the statement of Dr. T. McLayn in the *Lancet*, that, given at the commencement of the attack, it seems to arrest the course of the malady as effectually as quinia cures an ague, or ipecacuanha a dysentery; and says it cures rheumatism much more effectually than ipecac does dysentery in his practice; also that relief of pain is always one of the earliest effects produced. So much is this the case as applied to salicylic acid, that in no instance where he has used this remedy has he had occasion to administer a single dose of opium or any other medicine of that class.

**Quinetum.**

Dr. H. J. Vinkhuysen, physician to the household of the king of the Netherlands, writes to *The Practitioner* of February, regarding his experience with the collective alkaloid from Peruvian bark, called quinetum. He has used it in one hundred cases, and formulates his conclusions as follows:

1. the only malarious disease in which quinetum cannot be employed in place of quinine is pernicious fever. Quinetum requires more time to act than quinine, and as rapidity of action is absolutely necessary in this disease, quinetum cannot be used in it as a substitute for quinine.

2. In all forms of pure malarial intermittent fever, quinetum has the same apyretic effect as quinine, but is less powerful, and acts more slowly. It must therefore be given in large doses and at longer intervals before the ague fit, than quinine.

3. Quinetum does not produce the unpleasant and even dangerous symptoms of quinine when given during the fit, and may be taken during the fit without causing any unpleasant feeling.

4. Quinetum never causes noises in the ear.

5. Persons who are liable to suffer from the toxic effects of quinine, and who therefore cannot take it without the greatest discomfort, can take quinetum without this unpleasant effect, and yet obtain a similar therapeutical result.

6. The influence of quinetum in chronic cases is greater than that of quinine.

7. The tonic action of quinetum is similar and perhaps even greater than that of quinine.

8. The action of quinetum in cases of masked or larval malaria, and especially in rheumatic affections due to malarious influences, is incomparably greater than that of quinine.

---

**The Oil Bath in Disease.**

Baths of various kinds, besides those of water, have in all ages and among all people, been employed for the promotion of health or purification. Some of them, however, have been sufficiently nasty to recommend them with persons who imagine that great virtue must belong to filthiness. *Mud* baths belong to this species. They were much used by the ancients. The mud and ooze of the sea shore and rivers were preferred. The Egyptians still employ baths of this kind for scrofula, scurvy, and certain constitutional affections. In Germany, at Franzenbad, a mud bath is made of a peculiar black, bog earth that that place is favored with, which is mixed with water. This is claimed to be serviceable in paralytic affections having a gouty origin, in general debility, and it renders the skin as soft as satin. In France, it has been found that a *dung* bath is good for rheumatism. The *oil* bath, however, has antiquity to give it authority. There is reason for thinking it may often be useful. In Oriental countries, it is a remedy against the plague and other diseases of a contagious character, but is far from being always efficacious.

For anointing purposes, it seems, in the early history of the Israelites, to have found great favor, and to have been imposed by the rights of religion. Smearing with fats or oil is not, to the modern ideas, a process to be desired. It looks like being dirty, and especially must it feel so. But there is a good deal of testimony in the latest practice of medicine, and recorded among the medical literature of the day, which shows that the free use of oil over the body of the sick has a wonderfully restorative effect. The sudden change it causes from disease to health is marvellous. This is effected by simply anointing with oil. Sometimes full baths have been resorted to, but rubbing over is sufficient. Olive oil has been most employed, because no doubt, it is easily obtained and less disagreeable than most other oils. We have known cosmoline and vaseline, two new oils of a jelly-like consistence and appearance, to have been brought into service for this purpose. They have the advantages at least that they never undergo chemical change, are free from any disagreeable smell, easily applied on account of their density, and will not readily run in rivulets over the skin; they thus avoid a consequence of great fluidity—the general greasing of everything in the neighborhood of the patient. Oil baths can be made more agreeable by aromatizing them with some of the fragrant volatile essences. Oils of cassia, cloves, nutmegs, and cedrat may be digested or dissolved simply in the oil that forms the base. The material will then be ready for medical use. A good time for its application is at bed time. Woolen shirts and drawers are convenient and useful. They will, of course, be thoroughly greased in time, but then they can be washed, and no harm to them will be done.

It may be interesting to append to the preceding observations on the bath, part of a communication from Dr. Knaggs, of London, addressed to the *London Lancet* in 1870, which tends to illustrate some of the effects of the oiling treatment. His remarks in the article deal only with the affections of infants, but they are equally if not more applicable to persons of larger growth. He speaks especially of atrophy, bronchitis, convulsions, diarrhœa, febrile disturbances generally, and indeed all the disorders of childhood which are accompanied by an unnatural state of the skin. The treatment consists in greasing with salad oil the whole surface of the body, from the crown of the head to the tips of the fingers and toes, the process being repeated every twelve, six, or even four hours, according to the urgency of the case. His theory of the action of oil is—*First*, that the skin function is completely and permanently restored. *Second*, the danger of reaction is avoided, for there is no sudden change of temperature, and the sheet of oil protects the surface from atmospheric influences. *Third*, it acts as a fuel food, and prevents waste of tissue. *Fourth*, it appears to exhilarate.

The most formidable affections, the writer says, will frequently show signs of abatement in from twenty minutes to four-and-twenty hours, though sometimes a longer time is required. In a case of *atrophy*, an infant was *in articulo mortis*. The mother said she had sent for the doctor for "satisfaction only." The child was oiled, and in twenty minutes began to look about it and took food. Its recovery in the course of a fortnight was complete. Several of such cases are referred to. A desperate attack of *bronchitis* is mentioned. Under the ordinary treatment, the child grew rapidly worse. It was then bathed all over with salad oil, and in twenty minutes there was a marked improvement in breathing. In a few hours the symptoms had quite subsided. Other cases are related. *Convulsions :* In these cases, the effect of oiling is sometimes truly surprising, the fit ceasing before the completion of the operation and not returning. When the symptoms preceding convulsion are noticed, oiling the patient wards off the attack. Several other diseases, including diarrhœa, are mentioned, all of which are equally benefited by the anointing medication.

---

**Burns.**

A most remarkable property has recently been discovered in bicarbonate of sodium, viz: its power of arresting the pain of a burn. A physician was called to a fireman who had received a burn, involving two-thirds of the face, both ears, and extending over the whole back of the neck to down between the shoulder-blades. Both hands were also severely burned, and the suffering was intense. Enough water was added to bicarbonate of sodium to make it of the consistence of a thin paste, which was smeared over the burnt surface ; cloths wet with a saturated solution of sodium bicarbonate, were also applied. The relief to the pain was prompt and permanent.

